# Prognostic factors in diabetes: Comparison of Chi-square automatic interaction detector (CHAID) decision tree technology and logistic regression

**DOI:** 10.1097/MD.0000000000031343

**Published:** 2022-10-21

**Authors:** Hae-Young Choi, Eun-Yeob Kim, Jaeyoung Kim

**Affiliations:** a Medical Device Development Center, Daegu-Gyeongbuk Medical Innovation Foundation, Daegu, Republic of Korea; b Research Institute for Skin Image, Korea University College of Medicine, Seoul, Republic of Korea; c Core Research & Development Center, Korea University Ansan Hospital, Gyeonggi-do, Republic of Korea.

**Keywords:** diabetes, CHAID, decision tree, prediction

## Abstract

This study aimed to develop a diabetes prediction model. The model performance was compared with logistic regression, and the decision tree Chi-square automatic interaction detection (CHAID) was used to predict diabetes.

In total, 3233 patients were included in the analysis. Of these, 589 patients with diabetes and 2644 patients without diabetes were included after analyzing the study sample from the Korean Genome and Epidemiology Study (KoGES)-8 data. In this study, Diabetes Mellitus (DM) diagnosis prediction was compared with logistic regression and prediction through machine learning (ML) using the CHAID decision classification tree. We performed statistical analysis using the CHAID method with International Business Machine (IBM) statistical program SPSS®.

We performed logistic regression analysis to predict the classification of diabetes accurately, and the total classification accuracy of the analysis was 81.7%, and the CHAID decision tree classification accuracy was 81.8%. A diabetes diagnosis decision tree was created, which included seven terminal nodes and three depth levels. This analysis showed that a blood pressure problem and hospital visits were the most decisive variables at the time of classification, and two risk levels were created for diabetes diagnosis.

The suggested method is a valuable tool for predicting diabetes. Patients who visit the hospital because of blood pressure problems are more likely to develop diabetes than under-treating hyperlipidemia. The diabetes prediction model can help doctors make decisions by detecting the possibility of diabetes early; however, it is impossible to diagnose diabetes using only the model without the doctor’s opinion.

## 1. Introduction

The world’s diabetes population is estimated to be more than 400 million adults, and by 2045, it is expected that among 700 million people, more than one in 10 adults will have diabetes.^[1]^ According to the Organization for Economic Co-operation and Development (OECD) health statistics, an average of 22.7 deaths per 100,000 people were due to diabetes.^[2]^ In Korea, the prevalence of diabetes is estimated to be 1 in 7 adults aged over 30 years, and approximately 50 million people are being treated for diabetes.^[3]^ If diabetes can be predicted, preventive treatment, diabetes-related complications, and medical expenses will be reduced, and quality of life will improve.^[4]^ Since 2015, the Obama administration has implemented the Precision Medical Cohort Program (PMI-Cohort Program), a cancer genome discovery and clinical application, and promoted the transition from treatment-oriented to prevention-oriented medical systems.^[5]^

Diabetes mellitus (DM) includes type 1 diabetes, which occurs when the pancreas does not secrete insulin, and type II diabetes, which occurs when insulin is secreted but insulin resistance is increased.^[3]^ Diabetes is a chronic disease accompanied by complications, including retinopathy, nephropathy, and neuropathy. Moreover, it causes multiple risks, such as cardiovascular disease, and requires continuous management, treatment, and lifestyle changes.^[6]^ In addition, diabetes is a major risk factor for cardiovascular disease, and diabetic complications tend to increase the burden of patients’ medical expenses, increase socioeconomic losses, and have higher mortality rates than patients with other diseases.^[7]^ Many studies have assessed risk scores to select patients at high risk of diabetes.^[8–12]^ Diabetes is most effective in the prevention and management of high-risk groups before occurrence is very important. Therefore, it has become more important to identify appropriate criteria to predict and mediate the onset of diabetes in advance.^[13,14]^

Artificial intelligence/machine learning (AI/ML) is called “learning from data” or “data-driven algorithm” data-driven algorithms, which find the classification and clustering rules inherent in the data by applying feature representation and learning algorithms to the collected data.^[15]^ Recently, modeling research using ML technology based on electronic medical record (EMR) big data analysis has progressed with development and showed almost similar predictions to clinical diagnoses.^[16]^ Based on the demographics and clinical factors of patients with diabetes, we have also developed a diabetes prediction self-measurement model that is easily accessible and can be used by the general public.

This study compared and analyzed the logistic regression analysis among the traditional statistical methods for predicting diabetes occurrence and the CHIAD model among the data mining prediction methods. In addition, we analyzed classification and predictive models through the interaction effect and non-linearity of explanatory variables affecting the occurrence of diabetes with CHAID.

## 2. Methods

In this study data, the Korean Genome and Epidemiology Study (KoGES-8) of a community-based cohort (Ansan, Anseong) of the (KoGES) were used. The KoGES data is composed of “population-based” data from adults aged 50 or older and “gene-environment” to identify risk factors for genetic-environmental interactions in chronic diseases at the Department of Genetic Mechanics at the Korea Disease Control and Prevention Agency (KDCA). A total of 3233 patients were included. Of which 589 patients with diabetes and 2644 patients without diabetes were included by cleaning the study sample from the KoGES-8 data. For this study, data were received and analyzed according to the online procedure after approval by the institutional review board of Korea University Medical Center (KUMC IRB-2020AS0124).

Chi-square automatic interaction detection (CHAID) classification tree: The reason for diabetes diagnostic prediction modeling using a CHAID decision tree is a graphic representation of a series of decision rules. Beginning with a root node containing all the cases, the tree branch is divided into different child nodes containing case subgroups. The criterion for partitioning (or branching) was selected after reviewing all possible variables of all available predictive variables. In the terminal node, a grouping of cases is obtained; thus, possible cases are homogeneous in relation to the values of the dependent variables.^[16–18]^ This algorithm determines how to optimally combine categorical or continuous variables to predict binary results based on “if-then” logic by dividing each independent variable into mutually exclusive subsets based on data homogeneity. In this study, the response variable was the presence or absence of diabetes diagnosis. Statistical analysis using the CHAID method was performed using the CHAID node included in the International Business Machine (IBM) statistical program (SPSS).

### 2.1. Statistical analysis

For demographic and clinical characteristics, we evaluated differences between groups using chi-square tests for categorical variables or Fisher exact tests, and Mann–Whitney *U* test or Student *t*-test for continuous and ordered variables, if appropriate. Discrete variables were expressed as number (percentage) and continuous variables as average (mean) and standard deviation (SD). General DM diagnosis prediction was compared with logistic regression and prediction through ML using the CHAID decision classification tree. The statistical significance level was set on 0.05. IBM Statistical Package for the Social Sciences (SPSS) program ver. 25.0 (IBM Corp., Armonk, NY, USA) was used for data analysis.

## 3. Results

The characteristics of the study population are summarized in Table 1. The mean age was 68.0 ± 8.0 for the non-diabetes group and 70.0 ± 8.0 for the diabetes group, indicating statistical significance (*P* < .001). Waist size was 89.5 ± 8.9 cm for the patients without diabetes and 92.0 ± 9.0 cm for the patients with diabetes, indicating a significant difference in waist size between the two groups (*P* < .001). The weight was 60.1 ± 10.6 kg for the patients without diabetes and 62.0 ± 10.5 kg for those with diabetes, indicating a statistical significance (*P* < .001). In the “no drinking” group, out of 1532 patients, 58.0% did not have diabetes, whereas out of 344 patients, 58.4% had diabetes. In the “stop drinking” group, out of 214 patients, 8.1% did not have diabetes, and out of 63 patients, 10.7% had diabetes. In the “drinking” group, out of 897 patients, 33.9% did not have diabetes, and out of 182 patients, 30.9% had diabetes, indicating an insignificant difference between the two groups (*P* = .076). In the “nonsmoker” group, out of 1800 patients, 68.1% did not have diabetes, and out of 396 patients, 67.2% had diabetes. In the “nonsmoking (past smoking)” group, out of 555 patients, 21.0% did not have diabetes, and out of 132 patients, 22.4% had diabetes. In the “smoking” group, out of 289 patients, 10.9% did not have diabetes, and out of 61 patients, 10.4% had diabetes, indicating an insignificant difference between the two groups (*P* = .721).

**Table 1:** DM diagnosis and characteristics of the study population.

Variables		Not have diabetes		Diabetes		*X*^2^/Z**	*P*
		N	**%**	N	**%**		
Sex	Man	1114	42.1	245	41.6	0.057	.811
	Woman	1530	57.9	344	58.4		
Age		68.0	8.00	70.0	8.00	–3.616	.000
Waist size		89.5	8.9	92.0	9.0	–5.999	.000
Hip size (cm)		94.6	6.3	95.2	6.5	–1.635	.102
Height (cm)		157.1	9.3	156.9	9.3	–0.609	.542
Weight (cm)		60.1	10.6	62.0	10.5	–3.921	.000
Drink	No	1532	58.0	344	58.4	5.145	.076
	No (stop)	214	8.1	63	10.7		
	Yes (drinking)	897	33.9	182	30.9		
Smoke	No	1800	68.1	396	67.2	0.654	.721
	No (stop)	555	21.0	132	22.4		
	Yes (smoking)	289	10.9	61	10.4		
Exercise*	No	2037	77.1	450	76.4	0.122	0.727
	Yes	606	22.9	139	23.6		

* Exercise is defined as regular exercise enough to make the body sweat.

** *X*^2^ Chi-square test/*Z* Mann–Whitney test.

### 3.1. Diabetes and physical factor concern

Diabetes and physical factors are shown in Table 2. For the increase in water intake, for the “no” group, out of 2156 patients, 81.6% did not have diabetes, and out of 455 patients, 77.4% had diabetes, whereas for the “yes” group, out of 485 patients, 18.4% did not have diabetes, and out of 133 patients, 22.6% had diabetes, indicating a statistical significance (*P* = .018). For weight gained or lost over the past month, in the “gain” group, out of 2329 patients, 89.9% did not have diabetes, and out of 491 patients, 85.5% had diabetes. In the “loss” group, out of 263 patients, 10.1% did not have diabetes, and out of 83 patients, 14.5% had diabetes, indicating a statistical significance (*P* = .003).

**Table 2:** Diabetes and physical factor.

Variables		Not have diabetes		Diabetes		*X* ^2^ **	*P*
		N	**%**	N	**%**		
Water	No	2156	81.6	455	77.4	5.626	.018
	Yes	485	18.4	133	22.6		
Urine	No	2028	76.8	430	73.5	2.797	.094
	Yes	614	23.2	155	26.5		
Fatigue	No	1500	56.9	310	52.7	3.452	.063
	Yes	1135	43.1	278	47.3		
Weight	Gain	2329	89.9	491	85.5	8.982	.003
	Loss	263	10.1	83	14.5		

* Weight change over the past month, Water increase in intake.

** *X*^2^ Chi-square test.

### 3.2. Diabetes and other present disease concern

The concerns regarding diabetes and other diseases are listed in Table 3. In “visit a hospital (medical institution) with blood pressure problem (last 2 years),” for the “no” group, out of 1503 patients, 56.9% did not have diabetes, and out of 183 patients, 31.1% had diabetes, whereas for the “yes” group, out of 1137 patients, 43.1% did not have diabetes, and out of 406 patients, 68.9% had diabetes, indicating that visited the hospital due to blood pressure problem was statistically significant (*P* < .001). In “myocardial infarction treatment status,” for the “no” group out of 2620 patients, 99.1% did not have diabetes, and out of 574 patients, 97.5% had diabetes, whereas for the “yes” group, out of 24 patients, 0.9% did not have diabetes, and out of 15 patients, 2.5% had diabetes, indicating a statistical significance (*P* = .001). In the “congestive heart failure treatment status,” for the “no” group, out of 2644 patients, 100.00% did not have diabetes, and out of 588 patients, 99.8% had diabetes, whereas for the “yes” group, out of one patient, 0.2% had diabetes, indicating statistical significance (*P* = .034). In “coronary artery disease treatment status,” for the “no” group, out of 2565 patients, 97.0% did not have diabetes, and out of 556 patients, 94.4% had diabetes, whereas for the “yes” group, out of 79 patients, 3.0% did not have diabetes, and out of 33 patients, 5.6% had diabetes, indicating a statistical significance (*P* = .002). In “hyperlipidemia treatment status,” for the “no” group, out of 2310 patients, 87.4% did not have diabetes, and out of 460 patients, 78.1% had diabetes, whereas for the “yes” group, out of 334 patients, 12.6% did not have diabetes, and out of 129 patients, 21.9% had diabetes, indicating a statistical significance (*P* < .001). In the “renal treatment status,” for the “no” group, out of 2627 patients, 99.4% did not have diabetes, and out of 580 patients, 98.5% had diabetes, whereas for the “yes” group, out of 17 patients, 0.6% did not have diabetes, and out of 9 patients, 1.5% had diabetes, indicating statistical significance (*P* = .030).

**Table 3:** Diabetes and other present disease concern.

Variables		Not have diabetes		Diabetes		*X*^2^/t**	*P*
		N	**%**	N	**%**		
Osteoporosis	No	2074	78.5	458	78.2	0.040	0.842
	Yes	567	21.5	128	21.8		
Fracture	No	2496	94.5	557	94.6	0.000	.983
	Yes	144	5.5	32	5.4		
Blood pressure	No	1503	56.9	183	31.1	129.091	.000
	Yes	1137	43.1	406	68.9		
Gastritis (gastric ulcer)	No	2557	96.7	575	97.6	1.328	.249
	Yes	87	3.3	14	2.4		
Allergic	No	2627	99.4	586	99.5	0.140	.708
	Yes	17	0.6	3	0.5		
Myocardial infarction	No	2620	99.1	574	97.5	10.857	.001
	Yes	24	0.9	15	2.5		
Thyroid disease	No	2558	96.7	569	96.6	0.031	.860
	Yes	86	3.3	20	3.4		
Congestive heart failure	No	2644	100.0	588	99.8	4.490	.034
	Yes	0	0.0	1	0.2		
coronary artery disease	No	2565	97.0	556	94.4	9.848	.002
	Yes	79	3.0	33	5.6		
Hyperlipidemia	No	2310	87.4	460	78.1	33.729	.000
	Yes	334	12.6	129	21.9		
Asthma	No	2595	98.1	582	98.8	1.251	.263
	Yes	49	1.9	7	1.2		
Peripheral vascular disease	No	2642	99.9	589	100.0	0.446	.504
	Yes	2	0.1	0	0.0		
Kidney	No	2627	99.4	580	98.5	4.730	.030
	Yes	17	0.6	9	1.5		
Lung cancer	No	2638	99.8	589	100.0	1.339	.247
	Yes	6	0.2	0	0.0		
Liver cancer	No	2643	100.0	589	100.0	0.223	.637
	Yes	1	0.0	0	0.0		
Colon cancer	No	2642	99.9	588	99.8	0.460	.497
	Yes	2	0.1	1	0.2		
Pancreatic cancer	No	2643	100.0	589	100.0	0.223	.637
	Yes	1	0.0	0	0.0		
Cerebrovascular disease	No	2567	97.1	567	96.3	1.099	.295
	Yes	77	2.9	22	3.7		
Urinary tract infection	No	2641	99.9	589	100.0	0.669	.413
	Yes	3	0.1	0	0.0		
Arthritis (degenerative, rheumatoid)	No	2432	92.0	544	92.4	0.094	.759
	Yes	212	8.0	45	7.6		
Gout	No	2628	99.4	587	99.7	0.614	.433
	Yes	16	0.6	2	0.3		
Pulmonary disease	No	2641	99.9	588	99.8	0.124	.725
	Yes	3	0.1	1	0.2		

Fracture (both simple and severe fractures) of the last 2 years data, Blood pressure problem (Hypertension or Hypotension): Visit to a hospital with blood pressure for the last 2 years and the treatment status for gastritis/gastric ulcer, allergic disease, myocardial infarction, thyroid disease, congestive heart failure, coronary artery disease, hyperlipidemia, asthma, peripheral vascular disease, kidney disease, lung cancer, gastric cancer, liver cancer, colon cancer, pancreatic cancer, cerebrovascular disease, arthritis (degenerative, rheumatoid), gout, and pulmonary disease.

** *X*^2^ Chi-square test/ *t* Mann–Whitney test.

### 3.3. Logistic regression

Logistic regression analysis was performed to predict the presence of diabetes in Table 4. The fit of the model was suitable, with *P* = .305 in the Hosmer and Lemeshow tests. All variables were entered into the model. Among the 2569 cases without diabetes, 2558 (99.6%) were accurately classified, and among the 569 cases with diabetes, 1.1% and six cases were accurately classified, with a total classification accuracy of 81.7%. Logistic regression analysis “weight gain or loss over the past month” was found to be Wald = 13.109, *P* < .001, and β = 0.516 showed a positive value, indicating that the higher the hyperlipidemia treatment status, the higher the probability of diabetes. “Visit the hospital due to blood pressure problem” was found to be Wald = 76.954, *P* < .001, and β = 0.931 showed a positive value, indicating a higher probability of having diabetes. The myocardial infarction treatment status” was found to be Wald = 7.839, *P* = .005, and β = 0.990 showed a positive value, indicating that the higher the myocardial infarction treatment status, the higher the probability of having diabetes. “Coronary artery disease treatment status” was found to be Wald = 4.461, *P* = .035, and β = 0.490 showed a positive value, indicating the higher the coronary artery disease treatment status, the more probability of having diabetes. “Hyperlipidemia treatment status” was found to be Wald = 12.244, *P* < .001, and β = 0.431 showed a positive value, indicating that the higher the hyperlipidemia treatment status, the higher the probability of diabetes. The estimated regression equation for diabetes prediction was calculated as follows: OR (diabetes) = -4.716 + 0.516*(weight gain/loss) + 0.931*(blood pressure visit hospital) + 0.990*(myocardial infarction-1) + 0.490*(coronary artery disease-1) + 0.431*(hyperlipidemia-1)

**Table 4:** Diabetes prediction logistic regression analysis.

Variables		*B*	S.E.	Wald	df	*P*	Exp (B)	95% confidence interval for EXP (B)	
								Low	High
1 step	Age	0.012	0.007	2.982	1	.084	1.012	0.998	1.025
	Waist	0.013	0.008	2.545	1	.111	1.013	0.997	1.030
	Weight	0.007	0.007	1.048	1	.306	1.007	0.993	1.022
	Water	0.180	0.117	2.369	1	.124	1.197	0.952	1.505
	Weight	0.516	0.143	13.109	1	.000	1.676	1.267	2.217
	Blood pressure (1)	0.931	0.106	76.954	1	.000	2.537	2.061	3.124
	Myocardial infarction (1)	0.990	0.354	7.839	1	.005	2.691	1.346	5.380
	Congestive heart failure (1)	23.268	4,0192.9	0.000	1	1.000	12740023970.2	0.000	
	Coronary artery disease (1)	0.490	0.232	4.461	1	.035	1.632	1.036	2.570
	Hyperlipidemia (1)	0.431	0.123	12.244	1	.000	1.538	1.208	1.957
	Kidney (1)	0.618	0.449	1.896	1	.169	1.855	0.770	4.468
	Constant	–4.716	0.669	49.762	1	.000	0.009		

Fracture (both simple and severe fractures) of pressure for the last 2 years, Blood Problem (Hypertension or Hypotension): Visit to the hospital with blood pressure for the last 2 years, Water: increased water intake, weight gain/loss, and the treatment status for myocardial infarction, congestive heart failure, coronary artery disease, hyperlipidemia, and kidney disease.

### 3.4. CHAID classification tree

**Figure 1.**
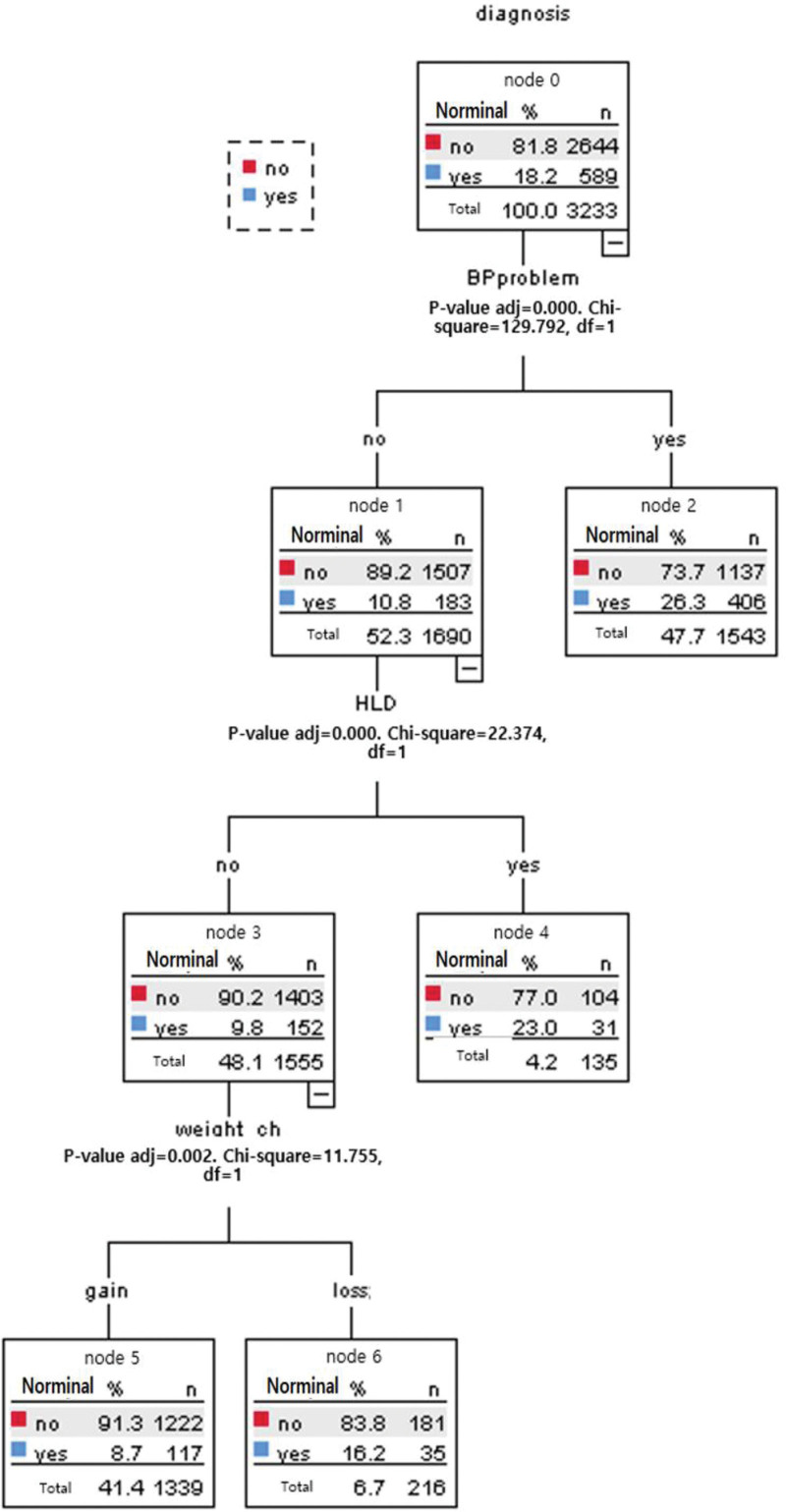
Tree created using the CHAID model (Chi-squared Automatic Interaction Detection) for diabetes.

In Figure 1, the analysis was conducted using the CHAID decision tree technique to obtain the best cutoff point for diabetes. Diabetes diagnosis was included as a dependent variable, and demographic (age, waist size, weight, drinking water, and weight change) and clinical (blood pressure problem, cardiac infarction, coronary artery disease, hyperlipidemia, and kidney disease) variables were used as independent variables. The maximum tree depth was three, with 100 minimum cases in the parent node and 50 minimum cases in the child node. The classification accuracy is 81.8%. A diabetes diagnosis decision tree was created, which included seven terminal nodes and three depth levels. This analysis showed that a blood pressure problem and hospital visits were the most decisive variables at the time of classification, and two risk levels were created for diabetes diagnosis. Visit to a hospital with a blood pressure problem (“no or yes”). Diabetes accounted for 10.8% of hospital visits for blood pressure problems (no). However, if the sub-node “hyperlipidemia” was treated, the diabetes probability increased to 23.0% (*X*^2^ = 22.374, *P* < .001), and visit to a hospital with blood pressure problems “no” was 10.8%. However, the probability of DM diagnosis increased to 16.2% in the case of sub-node hyperlipidemia “no” and weight loss (*X*^2^ = 11.755, *P* = .002). Visit to a hospital with blood pressure problems “yes” had a high diabetes diagnostic probability of 26.3% (*X*^2^ = 129.792, *P* < .001).

## 4. Discussion

The results of the study on patients diagnosed with diabetes are as follows: Based on the results of the logistic regression prediction, weight gain/loss and visits to a medical institution with blood pressure problems, myocardial infarction, coronary artery disease, and hyperlipidemia were the main factors in predicting diabetes. First, the CHAID model showed stage interactions between risk factors in step-by-step path identification to detect diabetes. The CHAID model was the strongest variable related to diabetes diagnosis, and “visit to a medical institution due to a blood pressure problem” was divided into the first level of the higher partitions than that of other variables. Second, among patients who did not visit a medical institution due to blood pressure problems, “hyperlipidemia treatment status” is an important predictor variable and has a 13.2% higher incidence of diabetes. Logistic regression also showed factors for detecting diabetes, but the CHAID model easily showed multilevel interactions by showing critical predictors in priority order. Therefore, the CHAID model is a tool that detects diabetes and supports clinician decisions, similar to the importance of logistic regression, a research method already known to detect diabetes.

Blood pressure problems were identified as the most important predictors of diabetes. Patients with blood pressure problems had a 16.3% higher incidence rate of diabetes than those without, which could eventually decrease diabetes if blood pressure is managed first. In the presence or absence of hyperlipidemia treatment linked to blood pressure problems, it is found that the patient’s weight gain or loss that is not currently being treated should be confirmed, which will help in clinical decision making by showing detailed diabetes prediction.^[19–21]^ These results showed a similar discriminant performance to that reported in other studies. Unlike general analysis, CHAID decision tree analysis can easily improve predictive ability using multivariate models, even in special situations, by analyzing the interactions of various variables and applying them to the entire population. Therefore, it is possible to detect individual cases showing unique behaviors within the entire research group that cannot be identified using the existing analysis methods. This result suggests that the incidence of diabetes can be easily predicted by excluding all clinical considerations for diabetes. However, there is a weakness in the diagnosis and judgment of clinical practice when considering the patient’s characteristics. This may help doctors prioritize the classification of patients according to their risk of developing diabetes.^[22]^

This study has some limitations. The study sample was limited to some regions and only Korean patients with diabetes were included. Factors for diabetes management were not considered. In addition, a predictive study was conducted on the entire disease spectrum of diabetes without distinguishing the detailed characteristics of diabetes. In the future, more large-scale randomized studies are required to clarify and specify the impact of the CHAID algorithm. Finally, in the CHAID model method, the number of terminal nodes tends to increase; however, information overload may occur because the number of target patients for each node is small. However, by predicting the occurrence of diabetes, it will be possible to reduce the incidence of diabetes, complications, and medical costs, and improve patient quality of life by predicting diabetes and other socially expensive and time-consuming diseases.

## 5. Conclusion

We identified blood pressure problems as the most important predictor of diabetes. Patients with blood pressure problems are more likely to develop diabetes than those without, and managing blood pressure first can eventually reduce diabetes. CHAID decision tree analysis analyzes the interaction of various variables. It applies to the entire population, making it easy to improve predictive ability using multivariate models even in particular situations. The suggested method can easily predict diabetes incidence within the study group, which conventional analytical techniques cannot identify. By predicting the onset of diabetes, it will be possible to reduce the incidence, complications, and medical costs of diabetes and improve patients’ quality of life by predicting diabetes and other socially expensive and time-consuming diseases. In the future, more extensive randomized studies to clarify and refine the impact of the CHAID algorithm.

## Acknowledgments

None.

## Author contributions

**Conceptualization:** Hae-Young Choi, Eun-Yeob Kim, Jaeyoung Kim.

**Data curation:** Hae-Young Choi, Eun-Yeob Kim, Jaeyoung Kim.

**Funding acquisition:** Jaeyoung Kim.

**Methodology:** Hae-**Young** Choi, Eun-Yeob Kim, Jaeyoung Kim.

**Project administration:** Jaeyoung Kim.

**Supervision:** Jaeyoung Kim.

**Writing—original draft:** Hae-Young Choi, Eun-Yeob Kim.

**Writing—review & editing:** Jaeyoung Kim.

## References

[1] International Diabetes Federation. IDF Diabetes Atlas. International Diabetes Federation. Available at: https://www.diabetesatlas.org [access date March 8, 2022].

[2] OECD. Health Care Utilisation. Available at: . http://stats.oecd.org/Index.aspx?DataSetCode=HEALTH_PROC. [access date March 9, 2022].

[3] Korean Diabetes Association. Diabetes fact sheet in Korea 2020. Available at: https://www.diabetes.or.kr/bbs/?code=eng_fact_sheet&mode=view&number=630&page=1&code=eng_fact_sheet. [access date March 8, 2022].

[4] Lee Y-h Kim DJ. Diabetes risk score for Korean adults. J Korean Diabetes. 2013;14:6–10.

[5] National Institutes of Health. All of Us Research Program. Available at: http://allofus.nih.gov [access date March 8, 2022].

[6] Punthakee Z Goldenberg R Katz P. Definition, classification and diagnosis of diabetes, prediabetes and metabolic syndrome. Can J Diabetes. 2018;42(Suppl 1):S10–5. 29650080 10.1016/j.jcjd.2017.10.003

[7] Kim SH Lee HB Jeon SW. Prediction of blood glucose in diabetic inpatients using LSTM neural network. J KIISE. 2020;47:1120–5.

[8] Bang H Edwards AM Bomback AS. Development and validation of a patient self-assessment score for diabetes risk. Ann Intern Med. 2009;151:775–83. 19949143 10.1059/0003-4819-151-11-200912010-00005 PMC3633111

[9] Ramachandran A Snehalatha C Vijay V. Derivation and validation of diabetes risk score for urban Asian Indians. Diabetes Res Clin Pract. 2005;70:63–70. 16126124 10.1016/j.diabres.2005.02.016

[10] Lindstrom J Tuomilehto J. The diabetes risk score: a practical tool to predict type 2 diabetes risk. Diabetes Care. 2003;26:725–31. 12610029 10.2337/diacare.26.3.725

[11] Aekplakorn W Bunnag P Woodward M. A risk score for predicting incident diabetes in the Thai population. Diabetes Care. 2006;29:1872–7. 16873795 10.2337/dc05-2141

[12] Gao WG Dong YH Pang ZC. A simple Chinese risk score for undiagnosed diabetes. Diabet Med. 2010;27:274–81. 20536489 10.1111/j.1464-5491.2010.02943.x

[13] Fukuda H Mizobe M. Impact of nonadherence on complication risks and healthcare costs in patients newly-diagnosed with diabetes. Diabetes Res Clin Pract. 2017;123:55–62. 27940390 10.1016/j.diabres.2016.11.007

[14] Blomster JI Woodward M Zoungas S. The harms of smoking and benefits of smoking cessation in women compared with men with type 2 diabetes: an observational analysis of the ADVANCE (Action in Diabetes and Vascular Disease: Preterax and Diamicron modified release Controlled Evaluation) trial. BMJ Open. 2016;6:e009668. 10.1136/bmjopen-2015-009668 PMC4716176 26747037

[15] Woo YC Lee SY Choi W. Trend of utilization of machine learning technology for digital healthcare data analysis. Electr Telecommun Trends. 2019;34:98–110.

[16] Park MS. Artificial intelligence application cases and considerations in digital healthcare. J Korea Converg Soc. 2022;13:141–7.

[17] Lee EJ Jeong IS Woo SH. [Development of a diabetic foot ulceration prediction model and nomogram]. J Korean Acad Nurs. 2021;51:280–93. 34215707 10.4040/jkan.20257

[18] Jang JS Lee MJ Lee TR. Development of T2DM prediction model using RNN. J Digit Converg. 2019;17:249–55.

[19] Rodriguez AH Aviles-Jurado FX Diaz E. Procalcitonin (PCT) levels for ruling-out bacterial coinfection in ICU patients with influenza: A CHAID decision-tree analysis. J Infect. 2016;72:143–51. 26702737 10.1016/j.jinf.2015.11.007

[20] Zhang J Goode KM Rigby A. Identifying patients at risk of death or hospitalisation due to worsening heart failure using decision tree analysis: evidence from the Trans-European Network-Home-Care Management System (TEN-HMS) study. Int J Cardiol. 2013;163:149–56. 21726908 10.1016/j.ijcard.2011.06.009

[21] Gan XM Xu YH Liu L. Predicting the incidence risk of ischemic stroke in a hospital population of southern China: a classification tree analysis. J Neurol Sci. 2011;306(1-2):108–14. 21489563 10.1016/j.jns.2011.03.032

[22] Aviles-Jurado FX Leon X. Prognostic factors in head and neck squamous cell carcinoma: comparison of CHAID decision trees technology and Cox analysis. Head Neck. 2013;35:877–83. 22711263 10.1002/hed.23058

